# Salvage therapy with high dose Intravenous Immunoglobulins in acquired Von Willebrand Syndrome and unresponsive severe intestinal bleeding

**DOI:** 10.1186/2162-3619-3-15

**Published:** 2014-06-04

**Authors:** Massimo Cugno, Alberto Tedeschi, Simona Maria Siboni, Francesca Stufano, Federica Depetri, Franca Franchi, Samantha Griffini, Flora Peyvandi

**Affiliations:** 1 Department of Pathophysiology and Transplantation, University of Milan, Milano, Italy; 2 Medicina Interna, Fondazione IRCCS Ca’ Granda, Ospedale Maggiore Policlinico, Milano, Italy; 3 Unità Operativa di Allergologia e Immunologia Clinica, Fondazione IRCCS Ca’ Granda, Ospedale Maggiore Policlinico, Milano, Italy; 4 Angelo Bianchi Bonomi Haemophilia and Thrombosis Centre, Fondazione IRCCS Ca' Granda Ospedale Maggiore Policlinico, and Luigi Villa Foundation, Milan, Italy

**Keywords:** Acquired von Willebrand syndrome, Intravenous immunoglobulin, Gastrointestinal bleeding

## Abstract

A 91-year-old woman affected with acquired Von Willebrand (VW) syndrome and intestinal angiodysplasias presented with severe gastrointestinal bleeding (hemoglobin 5 g/dl). Despite replacement therapy with VW factor/factor VIII concentrate qid, bleeding did not stop (eleven packed red blood cell units were transfused over three days). High circulating levels of anti-VW factor immunoglobulin M were documented immunoenzimatically. Heart ultrasound showed abnormalities of the mitral and aortic valves with severe flow alterations. When intravenous immunoglobulins were added to therapy, prompt clinical and laboratory responses occurred: complete cessation of bleeding, raise in hemoglobin, VW factor antigen, VW ristocetin cofactor and factor VIII levels as well as progressive reduction of the anti-VWF autoantibody levels.

## Introduction

Von Willebrand factor (VWF) is an adhesive glycoprotein synthesized by endothelial cells and megacaryocytes and released through a regulated pathway after storage in endothelial Weibel-Palade bodies and platelet alpha granules [1,2]. It circulates in plasma in large multimers and plays a pivotal role in primary haemostasis as it mediates adhesion of platelets to the subendothelium at sites of vascular damage [2]. The deficiency of VWF is associated with a hemostatic disorder and can be quantitative or qualitative [3]. It may be hereditary or acquired, with the hereditary form being one of the most common coagulation abnormalities in humans. In contrast, the acquired form is rare and generally occurs in individuals with no personal or family history of bleeding [3]. It is characterized by a prolonged bleeding time and variably low plasma levels of VWF and factor VIII, and is similar to the inherited form in terms of laboratory findings and clinical severity [4,5]. However, bleeding may be less predictable and more severe than in the congenital form. The pathophysiology underlying acquired Von Willebrand syndrome (VWS) is heterogeneous and none of the proposed mechanisms appears to be disease-specific [3-5]. Most patients have low plasma levels of VWF because of accelerated removal by three main mechanisms: 1) specific or non-specific autoantibodies that form circulating immune complexes with VWF and inactivate it (these complexes are cleared by Fc-bearing cells); 2) absorption of VWF onto malignant cell clones; 3) loss of high molecular weight VWF multimers under conditions of high shear stress [3-6]. Acquired VWS was first described in a patient with systemic lupus erythematosus in 1968 [7]. Subsequently, more than 300 cases have been reported, but the actual prevalence of the disease is underestimated because most patients do not bleed until they are exposed to major trauma or major invasive procedures and surgery [8].

Acquired VWS mainly occurs in patients with autoimmune, lymphoproliferative and myeloproliferative disorders, which account for 48–63% of cases; however, an association with solid tumors, cardiovascular disorders and hypothyroidism has also been described [9-13].

Treatment of acquired VWS is aimed to control acute bleeding episodes, to prevent bleeding when an invasive procedure is necessary, and when possible, to control the underlying disease [14].

Acute bleeding can be treated with desmopressin (DDAVP) and VWF/FVIII (factor VIII) concentrates or in unresponsive patients with recombinant factor VII [14]. Therapeutic approaches aimed to contrast autoantibodies include high-dose intravenous immunoglobulin, plasmapheresis, corticosteroids, and immunosuppressive drugs [14-16]. In particular, the effectiveness of intravenous immunoglobulin was demonstrated in an open-label crossover study in patients with acquired VWS associated with monoclonal gammopathy of undetermined significance of the IgG class but not of the IgM class [17].

We report a case of acquired VWS with mixed origin (autoimmune, due to anti-VWF IgM, and high shear stress, due to mitral and aortic valve abnormalities) whose severe and recurrent gastrointestinal bleeding was not controlled by replacement therapy alone, but promptly stopped after addition of high dose intravenous immunoglobulins. The hemostatic improvement was associated with a marked reduction in the clearance of VWF and FVIII as well as with a progressive decrease in the titer of anti-VWF autoantibody.

## Case description

A 91-year-old woman with a diagnosis of acquired VWS since the age of 86 was admitted to our hospital for severe and recurrent gastrointestinal bleedings.

The patient had a history of a previous right nephrectomy because of kidney stones at the age of 50, arterial hypertension, permanent atrial fibrillation, pulmonary chondromatous hamartoma diagnosed at the age of 72, previous colonic cancer treated with left hemicolectomy and locoregional lymphadenectomy at the age of 83. At the beginning, the patient presented a mild bleeding diathesis and was diagnosed with acquired VWS on the basis of coagulation abnormalities: factor VIII coagulant activity (FVIII: C) was 10% of normal pooled plasma, von Willebrand factor antigen (VWF Ag) was 7%, von Willebrand factor ristocetin cofactor (VWF:Rco) was <6%. The patient was treated with courses of factor VIII-rich VWF concentrate (FVIII concentrate/vWF-Hemate P, CLS Behring, Marburg, Germany). In the last 2 years, the patient underwent multiple hospitalizations for recurrent gastrointestinal bleedings due to intestinal angiodysplasias, diagnosed by double balloon enteroscopy. She was treated with blood transfusions, tranexamic acid, subcutaneous octreotide and FVIII/VWF concentrates (Haemate P). During one of her past hospitalizations, because of poor clinical response, Haemate P was also replaced by a high purity FVIII/VWF concentrate (Alphanate (Grifols, Los Angeles, CA, USA) without improvement of bleeding control. An unsuccessful attempt with atorvastatin was also done.

In the current hospitalization, on admission, the patient presented with abundant bright red blood in stool, blood pressure was 110/65 mmHg, pulse rate was 80 beats per minute (irregular), respiratory rate was 25 breaths per minute and oxygen saturation was 97% when breathing in air. On physical examination, bilateral lower limb edema was present. A grade 2/6 systolic murmur was heard at the mitral and aortic areas. Physical examination of chest and abdomen did not reveal any acute abnormality. Laboratory tests documented severe anemia with a minimum hemoglobin value of 5.0 g/dl and an increase in blood urea nitrogen (94 mg/dL). No additional alterations of routine laboratory tests were observed; in particular, activated partial thromboplastin time (aPTT), lactate dehydrogenase (LDH), haptoglobin and bilirubin were within the normal range and the Coombs test was negative. The electrocardiogram showed atrial fibrillation with normal intraventricular conduction and non specific repolarization abnormalities. The chest X ray was negative for parenchymal consolidations except for the known hamartoma.

During hospitalization, the patient was treated with infusion of octreotide (dose 25 μg/h) and replacement of factor VIII/VWF (Hemate P 2000 IU, corresponding to 2000 IU of FVIII and 4800 IU of VWF, every 6 hours). Although this therapy was continued, gastrointestinal bleeding did not stop, and a total of eleven bags of packed red blood cells were transfused over three days.

Contrast-enhanced CT scan of the abdomen and mesenteric vessel arteriography were performed without finding any single source of active or recent gastrointestinal bleeding.

**Figure 1:**
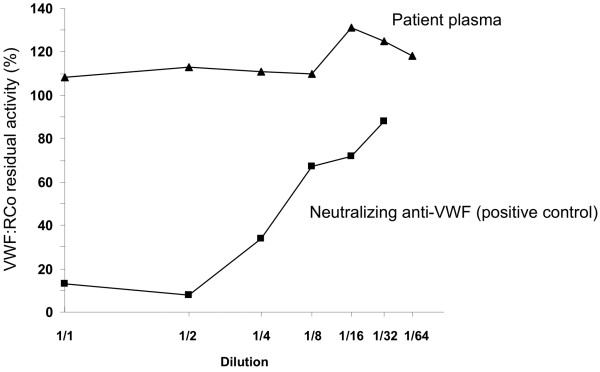
Von Willebrand factor ristocetin cofactor (VWF:RCo) residual activity of normal pooled plasma incubated with different dilutions of plasma from the patient with acquired von Willebrand syndrome (VWS) (Patient plasma) and plasma from another patient with acquired VWS containing neutralizing anti-VWF autoantibodies (positive control).

**Figure 2:**
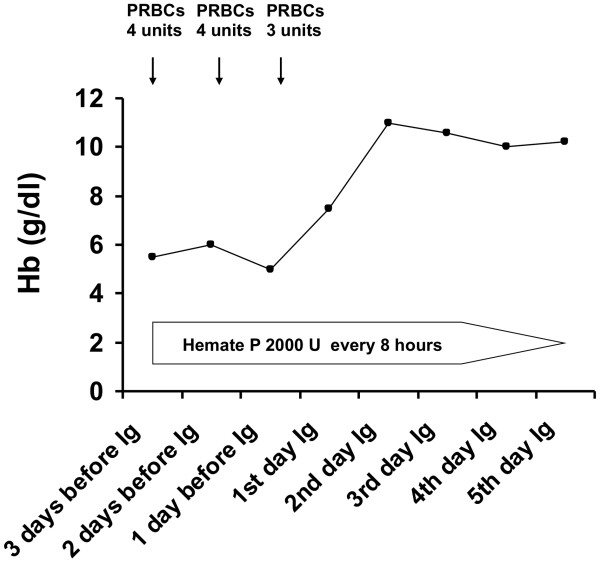
**Time course of haemoglobin concentration in peripheral blood of the patient with acquired von Willebrand syndrome before and after high dose immunoglobulin (Ig) infusion.** Units of packed red blood cells (PRBCs) which were transfused and replacement therapy with Hemate P are reported.

**Figure 3:**
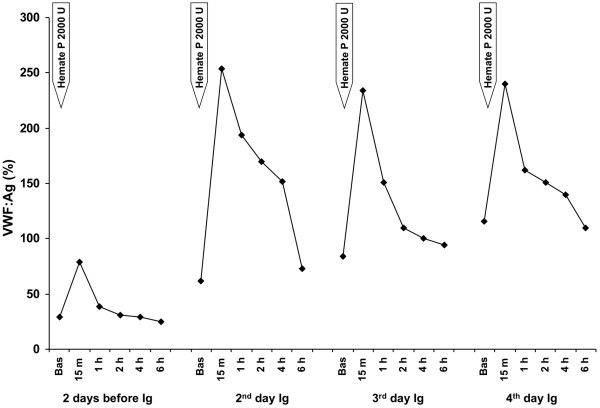
Kinetics of von Willebrand factor antigen (VWF:Ag) in plasma of the patient with acquired von Willebrand syndrome after replacement therapy with Hemate P. VWF:Ag levels were measured 2 days before and 2, 3 and 4 days after starting high dose intravenous immunoglobulin (Ig) infusion.

**Figure 4:**
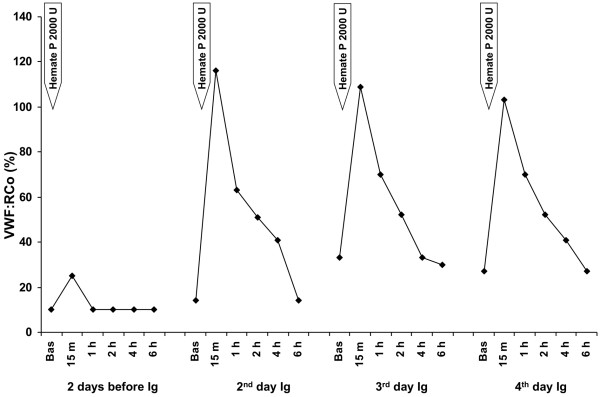
Kinetics of von Willebrand factor ristocetin cofactor (VWF:RCo) in plasma of the patient with acquired von Willebrand syndrome after replacement therapy with Hemate P. VWF:RCo levels were measured 2 days before and 2, 3 and 4 days after starting high dose intravenous immunoglobulin (Ig) infusion.

**Figure 5:**
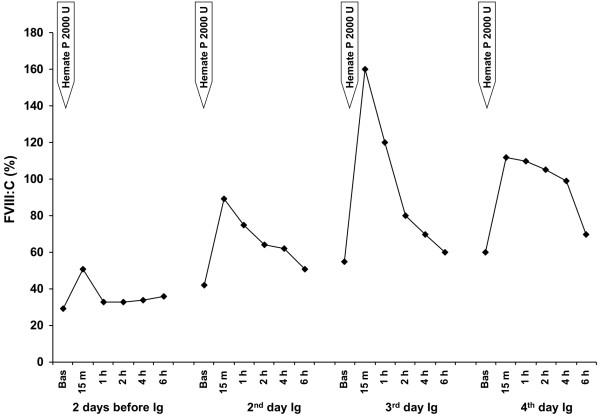
Kinetics of factor VIII coagulant activity (FVIII:C) in plasma of the patient with acquired von Willebrand syndrome after replacement therapy with Hemate P. Factor VIII:C levels were measured 2 days before and 2, 3 and 4 days after starting high dose intravenous immunoglobulin (Ig) infusion.

**Figure 6:**
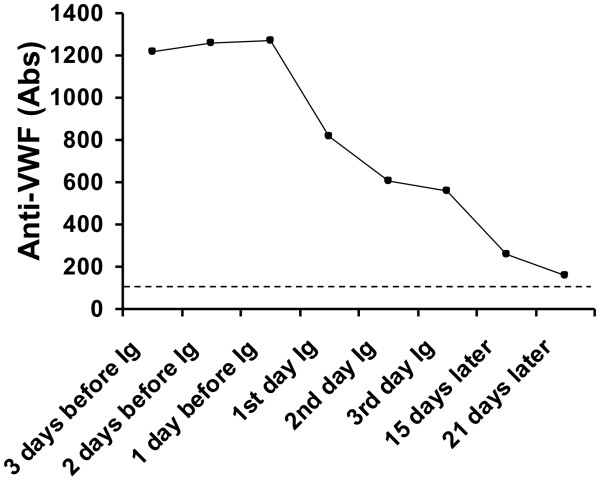
**Plasma levels of anti von Willebrand factor (anti-VWF) autoantibodies expressed as absorbance (Abs) at 492 nm in plasma from the patient with acquired von Willebrand syndrome before and after starting treatment with high dose intravenous immunoglobulin (Ig).** The dotted line represents the upper limit of the normal range.

An echocardiogram showed structural abnormalities of the mitral valve (fibrosis and partial retraction of the posterior leaflet and its support apparatus) and the aortic valve (diffuse calcifications of the cusps with partial coalescence of commissural margins); increased orthograde flow gradients (mitral mean gradient of 8 mmHg and peak aortic gradient of 19 mmHg). Sampling with color-Doppler showed flow abnormalities: mild aortic regurgitation with coaxial jet; severe mitral insufficiency with eccentric jet, dispersed along the lateral wall of the atrium (Coanda effect).Laboratory investigation by means of an immunoenzymatic method showed high circulating levels of anti-VWF IgM, whereas anti VWF IgG and IgA were negative. Briefly, purified VWF (kindly provided by doctor Friedrich Scheiflinger, Baxter, Vienna, Austria) was coated overnight onto microtitration plates and, after washing, the wells were coated with bovine albumin to avoid non-specific binding. After further washes, a 1:20 dilution of the plasma samples was added and incubated for 45 minutes at room temperature. After washing, the VWF-bound immunoglobulins were identified by means of class-specific mouse monoclonal antibodies, which were detected by means of peroxidase-conjugated anti-mouse immunoglobulin antibodies and revealed by orthophenylenediamine. The results were expressed as absorbance and referred to 20 normal subjects. The dilution curve of anti-VWF IgM from patient plasma was linear from 1500 to 200 absorbance (r = 0.999). The within- and between-assay coefficients of variation were less than 15%. By adaptation of the Bethesda method to VWF, the autoantibody against VWF was demonstrated to be non neutralizing (Figure 1). The presence of anti-VWF in patient’s plasma suggested an autoimmune origin of the bleeding disorder. For this reason, intravenous immunoglobulin (IVIg) was administered at a dose of 0.4 g/Kg/day for 5 days with good clinical and laboratory response (complete cessation of bleeding, raise in hemoglobin as well as in FVIII and VW:RCo plasma levels). In the 3 days before IVIg, due to the severe bleeding, the patient was transfused with 11 units of packed red blood cells (PRBCs) and her hemoglobin levels remained below 6 g/dl whereas, after IVIg, hemoglobin levels were stable at 10–11 g/dl without need of transfusion (Figure 2). IVIg had a dramatic effect on the kinetics of VWF:Ag, VWF:RCo and FVIII:C, inducing a 3- to 4-fold increase in their peak and recovery values after infusion of 2000 U of Hemate P (Figures 3, 4 and 5). Intravenous immunoglobulin also affected the levels of anti-VWF autoantibodies which progressively decreased reaching an 8-fold reduction 21 days later (Figure 6). After IVIg treatment, a therapy with prednisone (25 mg/day) and azathioprine (50 mg bid) was started.

At discharge, the patient was in a good general status, fecal occult blood test was negative and hemoglobin levels were 11.7 g/dl. The discharge therapy consisted in Hemate 2,000 IU tid, azathioprine 50 mg bid, omeprazole 20 mg bid and prednisone 25 mg/day.

## Discussion

Our case report clearly shows that it is possible to stop life-threatening bleeding in acquired VWS associated with anti-VWF autoantibody by the infusion of high dose IVIg in addition to replacement therapy with VWF/FVIII concentrate. In our patient with acquired VWS associated with IgM autoantibodies against VWF and high shear stress due to abnormalities of the mitral and aortic cardiac valves, IVIg strikingly modified the kinetics of VWF and FVIII with a 3 to 4 fold reduction in their clearance. Moreover, we observed a progressive reduction of anti-VWF autoantibody titer which started just after the beginning of IVIg therapy and continued afterwards, almost reaching the upper limit of normal 21 days later. Our data indicate that the anti VWF autoantibodies of our patient, albeit non neutralizing, markedly increase VWF/FVIII clearance, and high-dose IVIg may counterbalance this effect by their immunomodulatory properties.

IVIg is not recommended for routine use in the treatment of acquired VWS, according to evidence-based guidelines [18]. However, according to consensus by expert panels, IVIg may be considered one option among adjunctive therapies in urgent situations (eg, active bleeding or preoperatively) [18].

In an open crossover trial on 10 patients with acquired VWS associated with monoclonal gammopathy of undetermined significance (MGUS), Federici et al. demonstrated that the infusion of high-dose IVIg, in addition to DDAVP and plasma FVIII/VWF concentrate, induces transient (short-term therapy) and prolonged (long-term therapy) clinical and laboratory remission in IgG-MGUS but not in IgM-MGUS [17]. Maddox et al. reported successful cystectomy for relapsed transitional cell bladder carcinoma in a patient with acquired VWS and MGUS treated with a combined therapy of plasma exchange, high-dose IVIg and plasma FVIII/VWF concentrate [19]. Similarly, Eikenboom et al. obtained good clinical results in a patient who underwent cholecystectomy because of acute cholecystitis with perforation, using high-dose IVIg and plasma FVIII/VWF concentrate [20]. Lipkind et al. reported a pregnant woman with acquired VWS who underwent successful delivery with intravenous dexamethasone, FVIII/VWF concentrate and high-dose IVIg [21]. Kanakry and Gladstone reported the case of an 84-year-old man without a personal or family history of bleeding diathesis who underwent resection of a duodenal villous adenoma and developed a life-threatening gastrointestinal bleed. A diagnosis of acquired VWS was established and the patient was treated with high-dose IVIg obtaining the stop of bleeding within 48 hours. One month after IVIg, he was treated with rituximab for four weekly doses followed by eight doses of maintenance, and no further bleeding occurred [22].

Our results are in agreement with these observations and indicate the importance of the autoantibodies against VWF in the pathogenesis of the bleeding complications in acquired VWS by their marked effect on the kinetics of VWF and FVIII. The dramatic efficacy of high-dose IVIg in stopping the gastrointestinal life threatening bleeding further supports this view.

The prompt hemostatic response after IVIg administration may be due to rapid saturation of Fc receptors on cells of the mononuclear phagocyte system by the infused Ig, thus avoiding the clearance of autoantibody-VWF immunocomplexes [23]. Since the autoantibody of our patient has been demonstrated to be non neutralizing, it is conceivable that VWF of the circulating immunocomplex maintains its normal function. In addition, anti-idiotypic antibodies contained in IVIg may block the activity of pathogenic autoantibodies, thus contributing to the therapeutic effect observed in several antibody-dependent pathologies [24]. Finally, IVIg efficacy may be due to other non-exclusive actions on multiple pathways which, although poorly defined, can open new perspectives in the treatment of a growing number of autoimmune diseases [24-26].

The progressive decrease in the titer of anti-VWF autoantibody may be due to the effect of IVIg on human B cells. It was shown that IVIg promotes increased Fc receptor expression on plasma cells which may stimulate their sensitivity to apoptosis and have a direct impact on autoantibody production [27,28]. The immunosuppressive therapy with prednisone and azathioprine which was started after IVIg therapy, may have contributed to the decrease in the titer of anti-VWF autoantibody.

## Consent

Written informed consent was obtained from the patient for publication of this case report and any accompanying images. A copy of the written consent is available for review by the Editor-in-Chief of this journal.

## Abbreviations

aPTT: Activated partial thromboplastin time; DDAVP: Desmopressin (1-desamino-8-D-arginine vasopressin); FVIII:C: Factor VIII coagulant activity; IgG: Immunoglobulin G; IgM: Immunoglobulin M; IgA: Immunoglobulin A; IVIg: Intravenous immunoglobulin; LDH: Lactate dehydrogenase; MGUS: Monoclonal gammopathy of undetermined significance; qid: Quater in die (4 times a day); VWF: Von Willebrand factor; VWF Ag: Von Willebrand factor antigen; VWF:Rco: Von Willebrand factor ristocetin cofactor; VWS: Von Willebrand syndrome.

## Competing interests

The authors have no relevant conflicts of interest.

## Authors’ contributions

MC and AT wrote the manuscript. MC, AT, SMS, FD and FP participated in clinical management and discussion. FS, FF and SG performed laboratory tests. All authors read, discussed and approved the manuscript.
